# Fates of *Microcystis aeruginosa* Cells and Associated Microcystins in Sediment and the Effect of Coagulation Process on Them

**DOI:** 10.3390/toxins6010152

**Published:** 2013-12-30

**Authors:** Xiaoguo Chen, Huiyi Xiang, Yue Hu, Yang Zhang, Liao Ouyang, Meiying Gao

**Affiliations:** 1 Department of Environmental Science and Technology, Wuhan University of Technology, Wuhan 430070, China; E-Mails: xianghuiyi0925@163.com (H.X.); yue.hu@scilifelab.se (Y.H.); tayiyou@163.com (Y.Z.); ouyangliao_whut@126.com (L.O.); 2 State Key Laboratory of Virology, Wuhan Institute of Virology, Chinese Academy of Sciences, Wuhan 430071, China

**Keywords:** *Microcystis aeruginosa*, microcystins, coagulation, degradation, water bloom

## Abstract

During toxic *Microcystis aeruginosa* blooms, large amounts of cells can enter sediment through natural settlement, and coagulation treatment used to control water blooms can enhance the accumulation of cells. However, the current understanding of the fates of these cells and associated microcystins (MCs), as well as the effect of coagulation treatment on these factors, is limited. The results of the present study show that *Microcystis aeruginosa* cells in sediment were steadily decomposed under experimental conditions, and that they completely disappeared within 28 days. The major MCs released from settled cells were immediately degraded in sediment, and microbial degradation may be the main mechanism involved in this process. Coagulation treatment with PAC (polyaluminium chloride) + sepiolite can efficiently remove *Microcystis aeruginosa* cells from the water column and prevent their re-invasion. Furthermore, coagulation treatment with PAC + sepiolite had no significant effect on the release and decomposition of MCs and, thus, will not enhance the MCs pollution. However, coagulation treatment can accelerate the nutrient cycle by enhancing the settlement of cells. More attention should be paid to the effect on nutrient cycle when coagulation treatment is used for restoration of aquatic ecosystems.

## 1. Introduction

Cyanobacterial blooms, especially *Microcystis* blooms, have received worldwide attention due to their increase in occurrence and severity [1,2]. Massive growth of cyanobacteria could result in decreased water quality by releasing offensive taste and odor compounds [3]. More importantly, some cyanobacteria found in blooms can produce natural toxins [4], including microcystins (MCs), which are the most frequently encountered family of hepatotoxins. The growing concern regarding the acute and chronic effects of MCs has resulted in the World Health Organization setting a guideline value of 1 μg/L for microcystin-LR (MCLR) in drinking water [5]. Cyanobacterial cells can enter sediment through natural sedimentation [6,7,8,9], and this process does not occur exclusively at the end of the bloom, but, rather, throughout the entire bloom period [10]. Part of these cells may decay and decompose immediately after sedimentation [6,11], whereas more cells can withstand the environmental changes and survive for longer periods under stable conditions of low temperature and darkness [10,12]. However, the settled cells may also disappear from the sediment by decay or being grazed by protists [6,10,13,14]. Some cells even reinvade the water column in spring and serve as inoculum for the planktonic population, during which substantial loss of cells may occur by decay or programmed cell death [6,15]. Therefore, the abundance of cells in sediment often shows a temporal pattern, with a decline from spring to early summer owing to reinvasion, followed by an increase in late autumn due to settlement of pelagic cells [6,16].

Accumulation of large amounts of cyanobacterial cells in sediment may pose environmental problems. For example, the inoculation of resuspended cells may result in new blooms [6,17,18]. If large amounts of cells in the sediment decay and break down intensively, the sudden release of intracellular organic compounds and nutrients may deteriorate the water quality. Additionally, as MCs are usually contained in healthy cells [19], large amounts of intracellular MCs may enter the sediment together with cyanobacterial cells [8,10,20] and then be released into the surrounding water after the decay of cells [21]. Previous studies demonstrated that MCs released from cells are mainly reduced through dilution, adsorption, and biodegradation [20]. The biodegradation is considered as the main pathway for MCs elimination in the natural environment [21]. However, the relationship between release rate and degradation rate is still unknown. If the release of MCs is faster than the degradation, extracellular MCs will accumulate in sediment, and, hence, harm the benthic ecosystems [22] and even the water quality. Thus, it is important to understand the process that cyanobacterial cells and associated MCs are subjected to [10].

The presence of toxic cyanobacterial population in water bodies causes a serious threat for the health of humans [23]. To remove these cyanobacteria and associated MCs, many approaches including biological, chemical, mechanical, genetic, and environmental controls have been proposed [24,25,26]. Chemical coagulation process has been proved to be effective in eliminating cyanobacterial cells and intracellular MCs from drinking water sources [25,26,27,28,29,30]. Pan *et al.* [31] demonstrated that coagulation treatment can also be used for the removal of cyanobacterial blooms and the subsequent restoration of submerged vegetation in a whole bay (0.1 km^2^) experiment, suggesting that this technology might be effective in emergency treatment of cyanobacterial blooms or even the restoration of eutrophic water bodies. More recently, a combination of PAC (polyaluminium chloride) and modified clay was successfully used to restore a eutrophic lake through sinking cyanobacterial bloom and stabilizing phosphorus [32]. The treated lake has been shifted from a eutrophic/hypertrophic state to an oligo/mesotrophic state and kept this state for five years [32]. Although coagulation treatment gets rid of cyanobacterial cells from water column, it can not actually eliminate cyanobacterial cells from the water bodies. The cells are only transferred from the water column into the sediment. Hence, coagulation treatment may promote the accumulation of cyanobacterial cells in sediment, which could lead to new problems. Moreover, some coagulants may cause adverse effects on aquatic ecosystem because of their toxicity to aquatic organisms [33]. For example, if aluminum is used as coagulant in acidic conditions (pH < 6), ionic aluminum would be leached from insoluble forms and become toxic to many organisms, including microbes, plants, fish, and mammals [34]. Therefore, the potential effects of coagulation treatment on aquatic ecosystem function also deserve attention.

Previous investigations have described the dynamics of cyanobacteria and MCs in lake sediments under field conditions [6,7,10,21]. However, the details of this process have not been elucidated through experiments and the release and decomposition process of intracellular MCs after their entering the sediment is still unclear. Furthermore, although coagulation treatment can efficiently remove bloom algae and might be used for the restoration of aquatic ecosystems [31,32], its effects on the fates of settled cells and associated MCs have not been reported to date; thus, its safety is still unknown. This study was conducted to investigate the fates of sedimented *Microcystis* and associated intracellular MCs, as well as the effects of coagulation treatment on these compounds through laboratory simulation experiments.

## 2. Results

### 2.1. Natural Settlement and Coagulation Efficiency

*Microcystis aeruginosa* cells settled slowly without coagulant, with only 9.8% and 43.8% of cells settling to the sediment after 0.5 h and 8 h, respectively. The addition of coagulant significantly accelerated the settlement of *Microcystis aeruginosa* cells. The percentage of settled cells reached 69.4%, 99.3%, and 99.9% within 0.5 h after adding Sepiolite, PAC and PAC + Sepiolite, respectively, and more than 99% of cells were removed within 8 h in all coagulation treatments. 

### 2.2. Dynamics of *Microcystis aeruginosa* in Sediments

**Figure 1:**
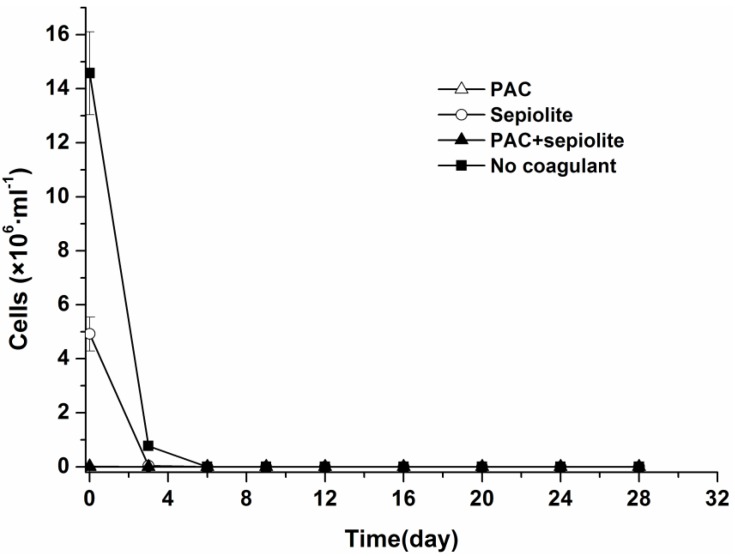
Dynamics of viable *Microcystis aeruginosa* cells in overlying water at 25 °C ± 0.5 °C in the presence of sediment. The error bars show the standard deviations.

The dynamics of viable cells in both overlying water and sediment were investigated by light microscopy after staining with trypan blue. Although *Microcystis aeruginosa* cells from the natural settlement treatment settled more slowly than those from coagulation treatments, no viable cells were detected in overlying water for any treatments at day six (Figure 1). 

**Figure 2:**
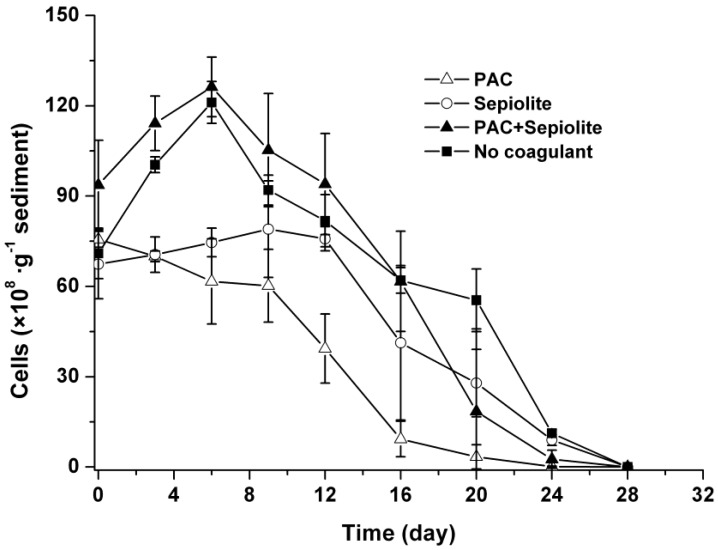
Dynamics of viable *Microcystis aeruginosa* cells in sediment at 25 °C ± 0.5 °C after coagulation treatment with PAC (250 mg/L), sepiolite (7 g/L), PAC (250 mg/L) + sepiolite (2.8 g/L), and no coagulant. The error bars show the standard deviations.

The changes in viable cells in sediments showed a similar trend in both natural settlement treatment and coagulation treatment with PAC + sepiolite (Figure 2). Specifically, the abundance of cells increased at the beginning of incubation, with a peak value being observed at day six. Thereafter, cells diminished steadily and no viable cells were detected at day 28. Although the patterns in the PAC treatment and sepiolite treatment were significantly different from that in the natural settlement treatment (*p* < 0.05), cells in these treatments also changed gradually and finally disappeared within 28 days. Moreover, there were fewer viable cells in sediment during the entire experiment for PAC treatment than for other treatments (Figure 2).

**Figure 3:**
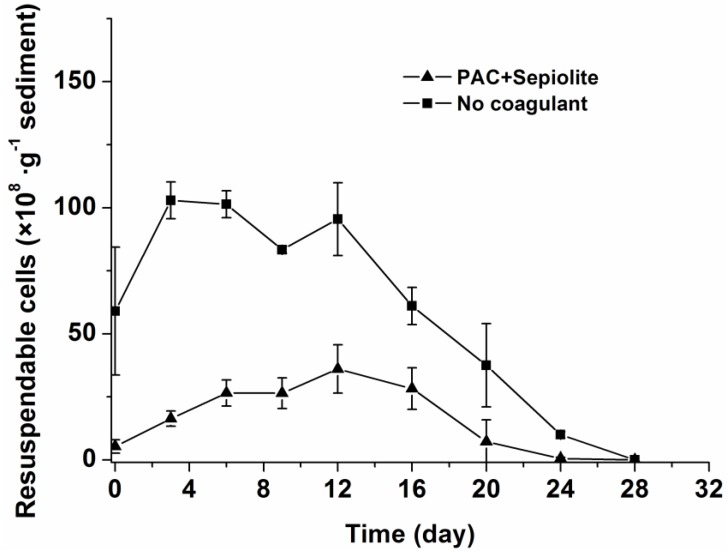
Dynamics of resuspendable cells in sediments at 25 °C ± 0.5 °C after coagulation treatment with PAC (250 mg/L) + sepiolite (2.8 g/L) and no coagulant. The error bars show the standard deviations.

The re-invasion potential of cells in sediments was further investigated for the natural settlement treatment and coagulation treatment with PAC + sepiolite. To accomplish this, sediment samples were suspended in water and then allowed to settle for five minutes, after which the cells suspended in the water phase (hereafter referred to as resuspendable cells) were counted by light microscopy. The dynamics of resuspendable cells in the sediment samples are shown in Figure 3. In the natural settlement treatment, the resuspendable cell numbers underwent the same changes as viable cells and the concentrations of resuspendable cells were only slightly lower than those of viable cells at most time points (Figure 2 and Figure 3). However, for the coagulation treatments (PAC + sepiolite), the concentrations of resuspendable cells were significantly lower than those of the viable cells in sediments throughout the experimental period. The resuspendable cells increased gradually at first, with the highest concentration being observed at day 12. Thereafter, the resuspendable cells decreased steadily and were completely gone at day 28. Although the same amounts of cells were added, the resuspendable cells in the coagulation treatment samples (PAC + sepiolite) were lower than those in the natural settlement samples.

### 2.3. Microcystin Release and Degradation

**Figure 4:**
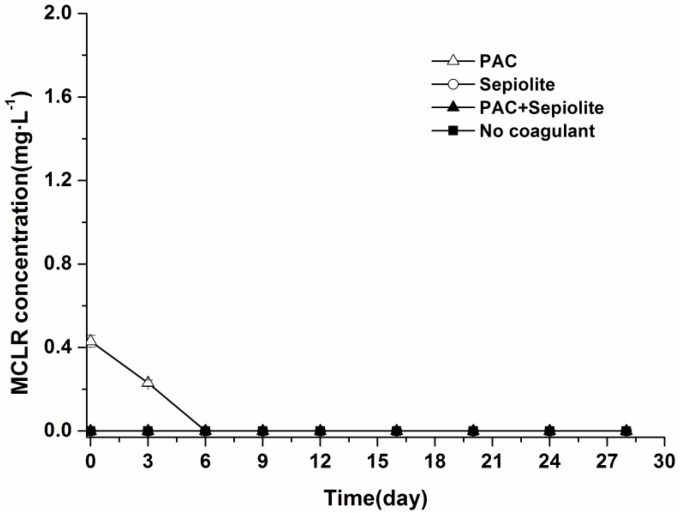
Dynamics of MCLR in overlying water at 25 °C ± 0.5 °C in the presence of sediment. The error bars show the standard deviations.

**Figure 5:**
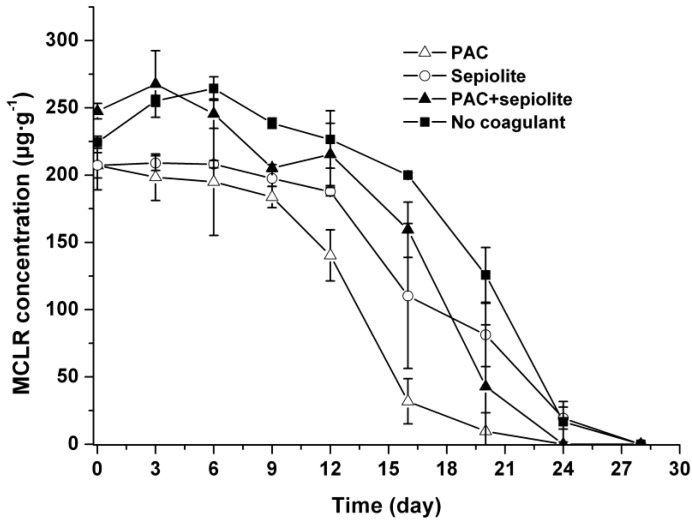
Dynamics of MCLR in sediment at 25 °C ± 0.5 °C after coagulation treatment with PAC (250 mg/L), sepiolite (7 g/L), PAC (250 mg/L) + sepiolite (2.8 g/L), and no coagulant. The error bars show the standard deviations.

During the incubation period, no MCLR were detected in the overlying water in any treatments other than the PAC treatment, in which a low level of MCLR (0.23 mg/L) was detected at day three (Figure 4). The dynamics of MCLR in sediment are shown in Figure 5. The curves of the MCLR concentration in various treatments showed a common trend (Figure 5). After about 6–12 days of lag phases, MCLR concentrations decreased gradually to below the detection limit at day 24 (PAC and PAC + sepiolite) and day 28 (natural settlement and sepiolite), respectively. During this course, no intermediate degradation product that had a UV spectrum similar to that of MCs was detected in the HPLC chromatogram. In addition, similar to the viable cells, the concentrations of MCLR in sediment were different for various treatments, and the concentrations in PAC treatment samples appeared to be lower than that in other samples. Nonparametric Spearman statistical analysis with data from all sediment samples showed that the concentration of MCLR in sediments was closely related to the *Microcystis aeruginosa* biomass in sediment (*r* = 0.93, *p* < 0.01, *n* = 36).

**Figure 6:**
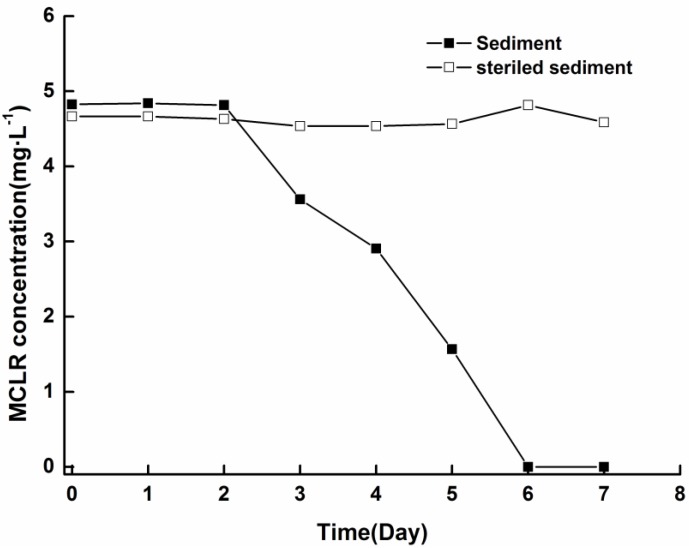
Biodegradation of soluble MCLR in sediment/aqueous system at 25 °C ± 0.5 °C.

To test whether MCs released from cells were decomposed by microbial community indigenous to the sediment, biodegradation experiments were conducted by adding soluble MCLR to sediment/aqueous system. The concentration of MCLR was effectively reduced to below the detection limits within six days, whereas no obvious reduction was observed in sterile control, indicating that losses of MCs in the experiments were not due to abiotic degradation (Figure 6). Furthermore, no intermediate degradation product that had a UV spectrum similar to that of MCs was observed in the HPLC chromatogram.

### 2.4. TN in Water Phase

**Figure 7:**
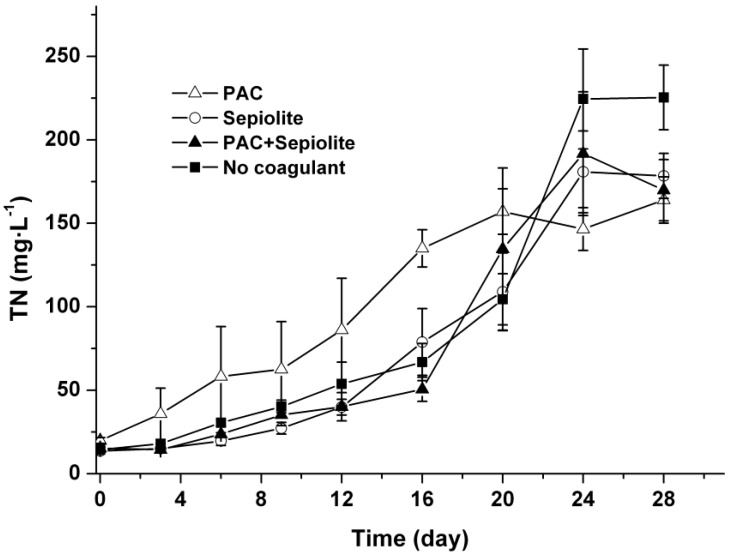
Profiles of TN in the water phase at 25 °C ± 0.5 °C after coagulation treatment with PAC (250 mg/L), sepiolite (7 g/L), PAC (250 mg/L) + sepiolite (2.8 g/L), and no coagulant. The error bars show the standard deviations.

The concentrations of TN in the water phase were monitored throughout the experiment using a TOC/TN analyzer after filtration through a 0.22 μm filter, and the results are shown in Figure 7. The TN concentrations in all treatments increased gradually from the beginning of the experiment to around day 24. No significant difference was observed among the TN profiles of various treatments (*p* > 0.05) except for the PAC treatment, in which the TN concentrations were higher than in other treatments during the first 20 days.

## 3. Discussion

Coagulation treatment has a strong potential for application in the restoration of aquatic ecosystems by removing bloom algae from water column [31,32], however, whether this process affects the fates of settled cells and thus causes new environmental problems is still unknown. Our results show that coagulation treatment removed *Microcystis aeruginosa* cells efficiently, and the combined use of PAC and sepiolite has higher removal efficiency compared with each coagulant alone. Furthermore, only a small part of viable cells was detected to be resuspendable in the coagulation treatment with PAC + sepiolite compared with the natural settlement treatment, indicating that the combined use of PAC and sepiolite can effectively prevent the cells from reinvading the water column. It is worth noting that distilled water was used in our experiment to suspend sediment during counting resuspendable cells. This may facilitate the dissolution of aggregate due to the low ionic strength in distilled water and thus make the cells immobilized in floc easy to be released. Therefore, the actual number of resuspendable cells in PAC + sepiolite treatment may be lower than that we observed. The less resuspendable cells is important for *in situ* control of bloom algae because the intense wind-induced mixing or bioturbation that often occurs in eutrophic water bodies can resuspend the sediment, resulting in the recruitment of benthic algae [35,36,37]. The effective removal of bloom algae and the inhibition of cells re-invasion by coagulation process may favor the rebuilding of submerged vegetation and subsequent restoration of eutrophic water bodies. 

Our results also suggest that coagulation treatment with PAC + sepiolite or sepiolite has no negative effect on *Microcystis aeruginosa* cells at the dose used in this study, as the cells number in sediment decreased gradually during incubation (Figure 2). In PAC treatment, however, a low level of MCs was detected in water phase during the early period of incubation (Figure 4), whereas no MCs were discovered in water phase after day three. Additionally, no massive decay of cells occurred during the whole experiments (Figure 2). These findings indicate that the lysis of cells and release of intracellular MCs might be due to the inadequate mixing of PAC at the beginning of coagulation experiment. These results differ from that of Han *et al.* [38], who found that alum treatment caused serious damage to *Microcystis ichthyoblabe* cells and resulted in the release of intracellular MCs no matter whether sediment was present or not. Although the exact reason for this discrepancy is still unknown, it is not likely due to coagulant dose, as a higher coagulant dose was adopted in present study (50.6 mg/mL as Al) than in above-described study (48 mg/mL as Al). The numbers of viable cells increased obviously in the early period of incubation for natural settlement treatment and coagulation treatments except PAC treatment. This might be attributed to the growth of *Microcystis aeruginosa* cells in sediment. During incubation, the sample bottles were brought out termly to detect the color change of sediment and overlying water. The exposure to light may promote the propagation of cells in the sediment surface and, thus, lead to the increase in cell numbers during the early period of incubation. In the mid-late period, however, this effect may begin to weaken as more cells decayed, hence the cell numbers commenced to decrease. A similar pattern appeared in the resuspendable cells in natural settlement treatment and PAC + sepiolite treatment. For the natural settlement treatment, the resuspendable cells changed synchronously with the viable cells in sediment and the resuspendable/viable cells ratio kept constant on the whole, suggesting that the variations of resuspendable cell numbers were attributed to the changes of viable cell densities. For the PAC + sepiolite treatment, however, the variations in resuspendable cells were not synchronized with the changes in the viable cells. Moreover, the resuspendable/viable cells ratio increased linearly from 0.05 at the beginning to 0.46 at day 16 and then decreased to 0.21 at day 24. These results suggest that the initial increase in resuspendable cells may be due to the increasing looseness of floc with time, rather than due to the growth of cells. In the later period of incubation, the resuspendable cells may be reduced owing to the significant decrease in viable cells. However, why the resuspendable/viable cells ratio decreased after day 16 was still unclear.

After coagulation treatment, massive MCs entering the sediment with intact cells may pose potential risk of MCs pollution. Our results show that coagulation treatment did not enhance the accumulation of MCs in sediment or lead to intense release of soluble MCs into water column. During incubation, the concentrations of MCs in sediment decreased steadily in the same manner as viable cells for all treatments. In addition, no MCs were detected in overlying water with the decay of cells, except in the PAC treatment, even though the total concentration of MCs in samples was as high as 250 μg/g sediment. These results imply that coagulation treatment has no significant effect on the release or decomposition of MCs in sediment. The synchronous change and high correlation between MCs concentration and cells number suggest that most MCs detected in sediment were contained in viable cells. Similar phenomenon was also demonstrated in a field investigation by Ihe [6]. As the MCs concentration (250 μg/g) in sediment was far higher than the adsorptive capacity of the sediment [20], the decline of MCs was not likely due to the irreversible adsorption to sediment. Therefore, the MCs were likely decomposed immediately after released from cells and would not enhance MCs pollution. In the present study, however, no pre-concentration or clean-up processes were performed before analyzing the MCs in the water phase due to the limited sample volume. As a result, it is still unconfirmed whether there was low level of MCs (less than 0.05 mg/L) entering the water column during treatment, even though no MC was detected in the overlying water. Since low-level prolonged exposure to MCs can also pose a risk to the environment and human health [23], the release and persistence of low levels of MCs deserves further investigation.

Our results indicate that biodegradation may be the major detoxification process of MCs released from cells in sediment. The biodegradation of soluble MCLR in sediment (Figure 6) argues that the MCs released from settled cells are likely degraded by MC-biodegrading bacteria indigenous to sediment. As biodegradation is a major process for natural elimination of MCs and the ability to degrade MCs is widely spread in water bodies, with abundance of MC-degrading bacteria inhabiting the sediment [21,39], bacterial biodegradation may play an important role in the fate of MCs released from settled cells under field conditions. During the degradation of MCs, no byproducts with the characteristic UV spectrum of MCs were observed upon HPLC analysis, implying that the Adda residue in the MCs had been disrupted. As the Adda residue is critical for MC toxicity, disruption of the Adda residue indicates that the toxicity of MCs might decrease remarkably. These findings suggest that MCs may be detoxified through biodegradation promptly after released from settled cells in sediment.

During the bloom period, bloom algae can also enter the sediment through natural sedimentation and sedimentation rates up to several g/m^2^/d have been reported in reservoirs [10]. The high sedimentation rates have caused massive accumulation of cells in sediment, with cell concentrations as high as 3 × 10^10^ cells/L [6]. Our results show that the naturally settled cells decomposed steadily and completely disappeared at day 28, days after a lag phase of six days, and during which no massive die-off of cells was noted (Figure 2). This decomposition rate is faster than that reported by Schöne [15], who demonstrated that only 27% of *Microcystis* cells in natural sediments disappeared at day 21. This discrepancy may be partly attributed to the different forms of *Microcystis*, as the cultured cells (unicellular form) have been used in present experiment, whereas colonial strains were studied in the reference [15]. Another possible reason was the difference in incubation temperature used in their study (9–15 °C) and this study (25 °C), because low temperature may suppress the biodegradation of *Microcystis* cells [12]. Similar effect of temperature was also found in a field investigation by Ihle [6]. The steady decomposition of settled cells at high temperature implies that these cells may not persist for very long periods of time in sediment. This explains why no massive accumulation of cells is detected during water bloom periods although large amounts of cells settle into sediment [6,10]. 

With the decay of settled cells, nitrogen element was synchronously released into water phase, suggesting that organic nitrogen in cells such as protein and nucleic acid was released. These kinds of organic nitrogen may be further decomposed by microorganisms and finally mineralized to NH^+^-N in water bodies. To avoid disturbing the floc, the disolved oxygen in our experiments was not measured during incubation. It is likely that both oxic and anoxic microenvironments are present in the same bottle due to the decay of algae cells. Hence, we supposed that some of NH^+^-N might be oxidized to NO_3_^−^-N through nitrification during incubation. Although coagulation treatment has no significant effect on the release of TN (Figure 7), it brings massive cells into the sediment. The steady lysis of these cells and the release of nitrogen may accelerate the nutrient cycle in water bodies. Therefore, more attentions should be paid to the effect on nutrients cycle when applying coagulation treatment. Han *et al.* [38] found that coagulation treatment with alume has negative effect on cyanobacteria-lysing organisms and microcystin-degrading bacteria. However, the same effect did not occur in present study, because no obvious inhibition on the lysis of algae cells and the degradation of MCs were observed. One possible reason may be the high pH (initial pH > 7 in all treatments) in our experiments. In neutral to slightly acidic conditions (pH > 6), aluminum is locked in minerals and is consequently nontoxic, whereas the Al^3+^ ion may be leached with increase in pH and appear toxic to aquatic organisms [34]. Besides aluminum, MCs may also negatively affect aquatic ecosystems due to their toxicity to aquatic plants and animals [40]. Previous studies reported that toxic *Microcystis aeruginosa* can be grazed by some protists and mixotrophic golden algae [14,41,42], and the intracellular MCs may affect the grazer growth [41]. These results suggest that the intracellular MCs may affect the structure of aquatic communities. Giaramida *et al.* [43] found that exposure to MCs can drive changes in structure and physiology of bacterial communities in sediment. Whether the same effects occurred in our experiment is still unclear as the structure of aquatic communities was not detected. In the present study, no extracellular MCs accumulated massively in our experimental conditions owing to their fast degradation, therefore, the acute impact of MCs on aquatic organisms may be negligible. However, as low-level prolonged exposure to MCs may also pose a risk to organisms [23], the chronic effects of extracellular MCs on aquatic ecosystem also deserve attention. 

## 4. Experimental Section

### 4.1. *Microcystis aeruginosa*

*Microcystis aeruginosa* (PCC 7806) was kindly provided by the Freshwater Algae Culture Collection of the Chinese Academy of Sciences, and originally obtained from the Pasteur Culture Collection of Cyanobacteria in France. Cultures were grown in BG-11 medium (pH 7.1) and maintained at 25 °C with fluorescent lamps as a light source (1500 lx). The cultures were harvested through centrifugation (4000 rpm for 15 min) during the late exponential phase of growth. The pelleted cells were then washed three times, after which they were suspended with sterile distilled water.

### 4.2. Sediment Samples

Samples of surface sediment were collected from Lake Dianchi (Kunming, Yunnan, China), where heavy cyanobacterial blooms have frequently occurred during the past 20 years. Samples were collected using a stainless steel grab sampler in December 2006. The treatment and characteristics of the sediment samples have been described previously [44].

### 4.3. Experimental Design

The coagulation experiments were conducted in a jar test unit with six paddles. Three types of coagulants, PAC, sepiolite and PAC + sepiolite, were used in the coagulation experiments. Since the primary purpose of this experiment was to elucidate the fates of sedimented *Microcystis aeruginosa* and associated MCs after coagulation treatment, a high initial concentration of cells (9.2 × 10^7^ cells/mL) was used to simulate the processes that may occur in practical coagulation treatment. In addition, more coagulants (PAC 250 mg/L, sepiolite 7 g/L, PAC 250 mg/L + sepiolite 2.8 g/L) than which are used in practical coagulation (clay 0.1–2.5 g/L, less than 10 mg Al/L) were employed to efficiently remove this high concentration of cells [32,45]. To analyze the coagulation efficiency, coagulant was added to 0.4 L of algae suspension at the beginning of a 1-min rapid mix cycle (200 rpm) to mix coagulant with suspension sufficiently. The cell suspensions were then mixed slowly at 50 rpm for 10 min to form macro-floc, after which the resultant floc was allowed to settle. Subsequently, 0.5 mL of water was siphoned from 1 cm below the water surface after 0.5 and 8 hours, and the cells in the samples were then counted by light microscopy. 

**Figure 8:**
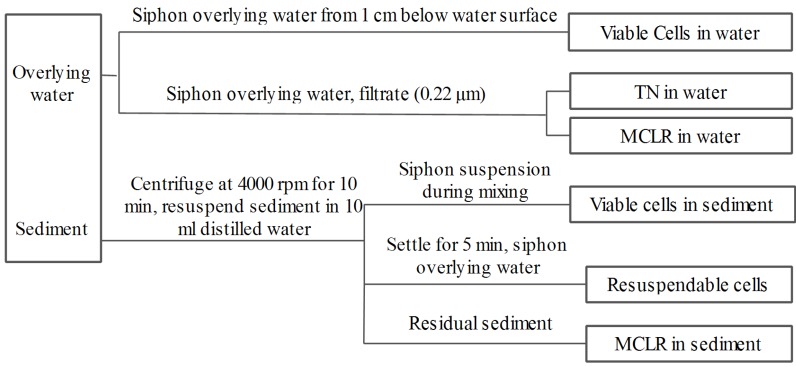
Flow chart of sampling procedure.

To investigate the fate of cells in the sediment, coagulant was also added to 0.4 L of algae suspension at the beginning of a 1-min rapid mix cycle (200 rpm). Then the cell suspensions were mixed slowly at 50 rpm for about 10 min. Before the end of slow mix cycle, the cell suspensions were distributed into nine bottles (40 mL per bottle) containing 0.4 g of sediment by siphon, and during which the cell suspensions were mixed with sediment by gentle shaking. The initial pH was detected by pH meter and was between 7 and 7.6 in all treatments. The bottles were then capped with cotton plugs and incubated in darkness without shaking at 25 °C ± 0.5 °C in an incubator. In the incubation period, the sample bottles were brought out of incubator termly to detect the color changes of sediment and overlying water. One bottle was selected for sampling at days 0 (after two hours of incubation), 3, 6, 9, 12, 16, 20, 24, and 28, and the sampling procedure was shown in Figure 8. During sampling, 0.5 mL of overlying water was siphoned from 1 cm below the water surface and the residual viable algae cells were measured by light microscopy. The remaining overlying water was then siphoned and filtered (0.22 μm), after which it was analyzed for total nitrogen (TN) using a MultiN/C2100 analyzer, as well as for MCLR by high performance liquid chromatography (HPLC). The residual sediments in the bottles were then centrifuged at 4000 rpm for 10 min and the pelleted sediment was then resuspended in 10 mL of distilled water. During mixing, 0.4 mL of the mixture was sampled to enumerate the residual viable cells in the sediment by light microscopy. The remaining portions of the mixture samples were allowed to settle for 5 min, after which 0.4 mL of water was sampled to count the viable cells by light microscopy. This value was then used to calculate the resuspendable cells in the sediments. The remaining mixture was used to measure the MCLR in sediment after further centrifugation. The fate of naturally settled cells in sediment was investigated as described above, except that no coagulant was added. All experiments were conducted in triplicate.

### 4.4. Biodegradability of Soluble MCs in Sediment

To verify whether soluble MCs can be decomposed by indigenous microbial community in sediments, biodegradation experiments were conducted according to the method previously reported [44]. The sediment (0.4 g) and 20 mL of sterilized distilled water were added to 25 mL brown glass bottles and mixed, followed by the addition of MCLR to produce a final concentration of 5 mg/L. The bottles were then capped with cotton plugs and incubated without shaking at 25 ± 0.5 °C. To detect MCLR, 0.5 mL of well-mixed sample was collected at different time intervals. A control experiment was performed, in which sediment and water was autoclaved at 121 °C three times before adding MCLR.

### 4.5. Viable Cells Assay

To distinguish living cells from dead ones, vital staining was carried out using Trypan blue solution (0.4%) [46]. Samples were mixed with coloring solution in the same proportion (1:1, *v*/*v*). After 5 min of incubation, the viable cells were counted in a hemocytometer under a light microscope. As viable cells have intact cell membranes, they do not take in dye. On the contrary, nonviable cells do not have intact membrane, hence they can take up dye and appear a distinctive blue color. 

### 4.6. MC Extraction and Analysis

Sediment samples were extracted for 15 min with 7 mL of 75% aqueous methanol under ultrasonic irradiation. The extraction was repeated three times, after which the combined supernatants were dried at 35 °C using a rotary evaporator (Laborta 4000-efficient, Heidolph, Schwabach, Germany) and then dissolved in 5 mL of 75% aqueous methanol for MCLR analysis. The MCLR concentrations in the extracted solutions and water samples were detected by HPLC as previously described [44].

### 4.7. Statistical Analysis

One-way repeated-measures ANOVA was performed using the statistical software package SPSS for Windows (SPSS 16.0, SPSS Inc., Chicago, IL, USA) to determine if there were significant differences between treatments. If a significant difference was detected, further comparison (Tukey’s method) was conducted to identify which specific treatments differed. *p* values < 0.05 were considered to be statistically significant.

## 5. Conclusions

The fates of *Microcystis aeruginosa* cells and associated MCs in sediment as well as the effect of coagulation treatment were studied by a simulation experiment. The results demonstrated that the settled cells were steadily decomposed within 28 days for all treatments, and high concentrations of TN were released into the water column during this period. Most MCs were degraded immediately after being released from the cells and microbial degradation may have been the major mechanism for this process. Coagulation treatment with PAC + sepiolite can not only efficiently remove *Microcystis aeruginosa* cells from the water column, but also prevent their re-invasion. In addition, it had no significant effect on the decomposition of MCs. During coagulation treatment, however, large amounts of cells were brought into the sediment, thus, the lysis of these cells and the release of these intracellular materials can accelerate the nutrient cycle. The results presented here have important implications for the management of water supplies contaminated with MC-producing cyanobacteria. 
